# Robust Entangled-Photon Ghost Imaging with Compressive Sensing

**DOI:** 10.3390/s19010192

**Published:** 2019-01-07

**Authors:** Jun Li, Wenyu Gao, Jiachuan Qian, Qinghua Guo, Jiangtao Xi, Christian H. Ritz

**Affiliations:** 1 National Lab of Radar Signal Processing, Xidian University, Xi’an 710071, China; wygao@stu.xidian.edu.cn (W.G.); jiachuanqian@sina.com (J.Q.); 2 School of Electrical, Computer and Telecommunications Engineering, University of Wollongong, Wollongong, NSW 2522, Australia; qguo@uow.edu.au (Q.G.); jiangtao@uow.edu.au (J.X.); critz@uow.edu.au (C.H.R.)

**Keywords:** entangled photons, compressive ghost imaging, quantum correlation

## Abstract

This work experimentally demonstrates that the imaging quality of quantum ghost imaging (GI) with entangled photons can be significantly improved by properly handling the errors caused by the imperfection of optical devices. We also consider compressive GI to reduce the number of measurements and thereby the data acquisition time. The image reconstruction is formulated as a sparse total least square problem which is solved with an iterative algorithm. Our experiments show that, compared with existing methods, the new method can achieve a significant performance gain in terms of mean square error and peak signal–noise ratio.

## 1. Introduction

Ghost imaging (GI) has raised increasing interest recently due to its wide applications ranging from biological sciences to security protocols [1,2]. In an entangled-photon GI system, the object reconstruction is based on two correlated optical beams, i.e., the object beam and the reference beam. The object beam emits light through an object, which is monitored by a bucket detector. The reference beam does not interact with the object and the light is monitored by a spatial-resolution detector. The first entangled-photon GI experiment was demonstrated by using entangled photons generated by spontaneous parametric down conversion (SPDC) [3,4,5,6,7]. Quantum imaging with entangled photons suffers from low-efficiency due to the low-flux of entangled photons, and it is also time-consuming. To solve this problem, compressive sensing (CS) was introduced into quantum GI [8,9,10], which greatly reduces the number of measurements and thereby the acquisition time [10,11,12]. Some investigations have been conducted to improve the quality of imaging in terms of peak signal–noise ratio (PSNR) and the mean square error (MSE). To improve the quality of reconstruction and reduce the number of samples in the GI system, many CS methods have been employed, which include Orthogonal Matching Pursuit (OMP) [13], Gradient Projection for Sparse Reconstruction (GPSR), etc. Compared to traditional quantum GI, the use of these algorithms could provide higher PSNR and lower MSE.

GI was originally performed using entangled-photon pairs [14], and then was realized with thermal light [15]. In thermal GI, a laser beam is used to illuminate an object, and a light is collected by a single-pixel bucket with no spatial resolution. By combining CS and GI, the spatial resolution of recovered images can beat the diffraction limit of GI [16]. Thermal GI has potential in practical applications [17], such as X-ray tomography [18], astronomy [19] and single-pixel imaging [20,21,22]. Compared with the thermal-light GI [23], quantum GI can obtain higher-visibility and imaging quality [24]. Quantum imaging can break the resolution limit of Rayleigh diffraction [25,26] to achieve super-resolution and strong anti-interference capability. However, the performance of the GI system can suffer from errors caused by the imperfection of optical devices and measurement. In particular, the spatial light modulator (SLM) which modulates the entangled photons in the GI system causes modulation errors. The errors caused by the imperfection of the optics devices were not considered in the literature.

In this work, we experimentally show that, by properly handling the errors caused by the imperfection of optical devices in an entangled-photon GI system, significant performance improvement can be achieved. Compressive GI is considered to reduce the data acquisition time. The image reconstruction is formulated as a sparse total least square (STLS) problem, that is solved using an iterative algorithm. Both simulation and experimental results are provided to demonstrate that a significant performance gain can be achieved by the proposed method in terms of PSNR and MSE, compared with existing compressive sensing-based methods.

## 2. Robust Ghost Imaging Based on STLS

**Figure 1:**
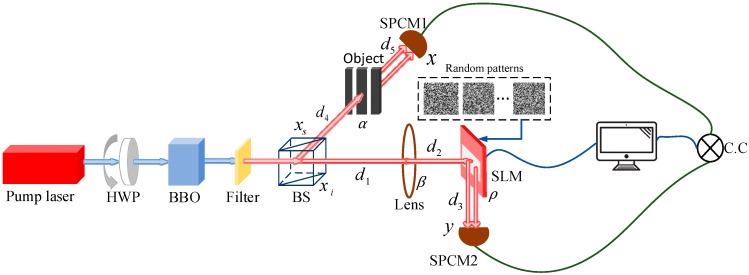
The experimental schematic of STLS quantum GI: HWP, half-wave plate; BBO, β-barium borate crystal; BS, beam splitter; Random patterns placed on a spatial light modulator (SLM); SPCM, single photon counting modules; C.C, coincidence measurement between SPCM1 and SPCM2.

We use a quantum compressive GI system where SPDC is used to generate photons, and entangled photons are used as the light source. The quantum GI setup [3,4,5,6,7] is shown in Figure 1.

SPDC is caused by random vacuum fluctuations, and the generation of entangled-photon pairs is random. The conversion efficiency of this process is extremely low. The bi-photon state can be represented as:

(1) $$|\psi \rangle =\iint dx_{s}dx_{i}\varphi \left(x_{s},x_{i}\right){\hat{a}}_{s}^{+}\left(x_{s}\right){\hat{a}}_{i}^{+}\left(x_{i}\right)|0,0\rangle$$

where |ψ〉 is the bi-photon state, $\varphi \left(x_{s},x_{i}\right)$ is the optical-field of the pump, ${\hat{a}}_{s}^{+}\left(x_{s}\right)$ and ${\hat{a}}_{i}^{+}\left(x_{i}\right)$ are the creation operators of signal light and reference light respectively. $x_{s}$ and $x_{i}$ are the positions of two optical paths at BS, respectively. x and y are the positions of photons at the cross-section of SPCM1 and SPCM2, and |0,0〉 is the vacuum state [27]. The coincidence count signal for the detectors SPCM1 and SPCM2 can be expressed by the fourth-order correlation function of optical field intensity as follows:

(2) $$\begin{array}{cc} C\left(x,y\right) & =\langle \psi |{\hat{E}}_{s}^{-}\left(x\right){\hat{E}}_{i}^{-}\left(y\right){\hat{E}}_{s}^{+}\left(x\right){\hat{E}}_{i}^{+}\left(y\right)|\psi \rangle \\ & ={\left|\langle 0|{\hat{E}}_{s}^{+}\left(x\right){\hat{E}}_{i}^{+}\left(y\right)|\psi \rangle \right|}^{2} \end{array}$$

where ${\hat{E}}_{s}^{+}\left(x\right)$, ${\hat{E}}_{i}^{+}\left(y\right)$, ${\hat{E}}_{s}^{-}\left(x\right)$, and ${\hat{E}}_{i}^{-}\left(y\right)$ are the positive and negative frequency part of the optical field operator at coordinate (*x*, *y*) [19]. The optical fields on the detection plane, which appear in the second line of (2), are given by:

(3) $$E_{s}^{+}\left(x\right)=\int dx_{s}h\left(x,x_{s}\right){\hat{a}}_{s}\left(x_{s}\right)$$

(4) $$E_{i}^{+}\left(y\right)=\int dx_{i}h\left(y,x_{i}\right){\hat{a}}_{i}\left(x_{i}\right)$$

The free-space propagation function of the object arm is:

(5) $$h\left(x,x_{s}\right)=\int d\alpha h\left(\alpha ,x_{s}\right)h\left(x,\alpha \right)$$

where h(x,α) is the free-space propagation function from α to x, and the free-space propagation function of the reference arm is expressed as follows:

(6) $$h\left(y,x_{i}\right)=\int d\beta d\rho h\left(\beta ,x_{i}\right)h\left(\rho ,\beta \right)h\left(y,\rho \right)L\left(\beta \right)$$

where the $L\left(\beta \right)=exp\left(i\pi /\left(\lambda f\right)\beta^{2}\right)$ is the optical transfer function of the lens. Here, f=100 mm is the focal length of the lens. Based on the Fresnel approximation, the two-photon amplitude can be expressed as:

(7) $$\begin{array}{c} \phi \left(x,y\right)=\int dx_{s}dx_{i}h\left(x,x_{s}\right)T\left(\alpha \right)h\left(y,x_{i}\right)(A_{i}\left(\rho \right)+\Delta A)+\Delta e\\ =\int dx_{s}dx_{i}d\alpha d\beta d\rho h\left(\alpha ,x_{s}\right)T\left(\alpha \right)h\left(x,\alpha \right)\\ \times h\left(y,\rho \right)(A_{i}\left(\rho \right)+\Delta A)h\left(\rho ,\beta \right)L\left(\beta \right)h\left(\beta ,x_{i}\right)\varphi \left(x_{s},x_{i}\right)+\Delta e \end{array}$$

where the free-space propagation function can be written as $h\left(x,x^{\prime }\right)\approx exp\left(i\pi /\left(d_{1}\lambda \right){\left(x^{\prime }-x\right)}^{2}\right)$, T(α) is the transmission function of the object, Δe represents measurement error, and $A_{i}\left(\rho \right)$ is a random pattern loaded onto the SLM. The bi-photon state generated by SPDC can be approximated by $\varphi \left(x_{s},x_{i}\right)\approx \delta \left(x_{s}-x_{i}\right)$ [28,29]. When the experiment satisfies the thin lens equation $1/\left(d_{1}+d_{4}\right)+1/d_{2}=1/f$ [30], and where $\left(d_{1}+d_{4}\right)/d_{2}=1.5$ is the theoretical magnification factor of our imaging system, where f is the focal length of the lens. Accordingly, two-photon amplitude is given by:

(8) $$\begin{array}{cc} \phi \left(x,y\right) & =\langle 0|{\hat{E}}_{s}^{+}\left(x\right){\hat{E}}_{i}^{+}\left(y\right)|\psi \rangle \\ & \propto \int dx_{s}dx_{i}d\alpha d\beta d\rho \exp\left(\frac{i\pi }{d_{4}\lambda }{\left(\alpha -x_{s}\right)}^{2}\right)T\left(\alpha \right)\exp\left(\frac{i\pi }{d_{5}\lambda }{\left(x-\alpha \right)}^{2}\right)\exp\left(\frac{i\pi }{d_{3}\lambda }{\left(y-\rho \right)}^{2}\right)\\ & \left(A_{i}\left(\rho \right)+\Delta A\right)\exp\left(\frac{i\pi }{\lambda f}\beta^{2}\right)\exp\left(\frac{i\pi }{d_{2}\lambda }{\left(\rho -\beta \right)}^{2}\right)\exp\left(\frac{i\pi }{d_{1}\lambda }{\left(\beta -x_{i}\right)}^{2}\right)\delta \left(x_{s}-x_{i}\right)+\Delta e \end{array}$$

Substituting (1), (3), (4) into (2), we have:

(9) $$\begin{array}{cc} C\left(x,y\right) & \propto |\int d\alpha d\beta d\rho \exp\left(\frac{i\pi }{\lambda \left(d_{1}+d_{4}\right)}{\left(\alpha -\beta \right)}^{2}\right)\exp\left(\frac{-i\pi }{\lambda f}\beta^{2}\right)\exp\left(\frac{i\pi }{d_{2}\lambda }{\left(\rho -\beta \right)}^{2}\right)\\ & {\exp\left(\frac{i\pi }{d_{5}\lambda }{\left(x-\alpha \right)}^{2}\right)\exp\left(\frac{i\pi }{d_{3}\lambda }{\left(y-\rho \right)}^{2}\right)Τ\left(\alpha \right)\left(A_{i}\left(\rho \right)+\Delta A\right)+\Delta e|}^{2} \end{array}$$

Then, the integrated coincidence signal becomes:

(10) $$\begin{array}{cc} C_{m}\left(x,y\right) & =\iint C\left(x,y\right)dxdy\\ & ={\iint \left|\phi \left(x,y\right)\right|}^{2}dxdy\\ & \propto \sum_{n=1}^{N}{\left|A_{m}\left(-\alpha_{n}\right)+\Delta A\right|}^{2}{\left|T\left(\alpha_{n}\right)\right|}^{2}+\Delta e \end{array}$$

where *m* ∈ 1, 2, …, *M* with *M* being the total number of measurements, and *n* ∈ 1, 2, ..., *N* with *N* being the number of pixels corresponding to the object. In (10), ${\left|A_{m}\left(-\alpha_{n}\right)+\Delta A\right|}^{2}$ can be rewritten as:

(11) $${\left|A_{m}(-\alpha_{n})+\Delta A\right|}^{2}={\left|A_{m}(-\alpha_{n})\right|}^{2}+2\times A_{m}(-\alpha_{n})\times \Delta A+{\left|\Delta A\right|}^{2}=A_{mn}+\Delta S_{mn}$$

where $\Delta S_{mn}=2\times A_{m}\left(-\alpha_{n}\right)\times \Delta A+{\left|\Delta A\right|}^{2}$ accounts for the errors caused by the imperfection of optical devices, and $A_{mn}={\left|A_{m}\left(-\alpha_{n}\right)\right|}^{2}$ is the (*m*,*n*)th element of the sensing matrix $A_{M\times N}$. Equation (10) can be rewritten in matrix form as:

(12) $$C_{M\times 1}=(A_{M\times N}+\Delta S)\times T_{N\times 1}+\Delta E$$

where $C_{M\times 1}$ is the measurement vector, and $A_{M\times N}$ is the sensing matrix, *M* is the number of samples. The N-dimensional unknown signal $T_{N\times 1}$ is a vector constructed by $T_{n}={\left|T\left(\alpha_{n}\right)\right|}^{2}$. ΔE is a measurement error vector constructed by Δe. In our method, pseudo-random measurement matrices $A_{M\times N}$ (*M < N*, *N* = 64 × 64) are selected as the sensing matrices. Given the initial parameters, we can obtain a complete sequence of numbers. The general form of the pseudo-random sequence can be written as $x_{n+1}=f\left(af^{-1}\left(x_{n}\right)\right)$ and $y_{n}=f\left(bf^{-1}\left(x_{n}\right)\right)$, where $f\left(x\right)={sin}^{2}x$, $a=2.01,b=100^{2}$. Then, we rearrange the sequence into a matrix with size M×N. This ill-posed [31,32,33] problem can be solved by exploiting the sparsity of the signal if $A_{M\times N}$ satisfies the restricted isometry property (RIP) [34,35].

Compared with TLS and other CS algorithms, STLS integrates the advantages of compressive sensing and TLS. On one hand, STLS can reconstruct images with only a small number of samples by exploiting the sparsity of the signal, i.e., STLS can deal with ill-posed problems while TLS cannot. On the other hand, STLS is able to handle the errors caused by both the imperfection of optical devices and measurement, thereby achieving robust GI and high imaging quality, which makes it superior to conventional CS algorithms as the conventional compressed sensing algorithm does not consider errors in the dictionary matrix.

**Figure 2:**
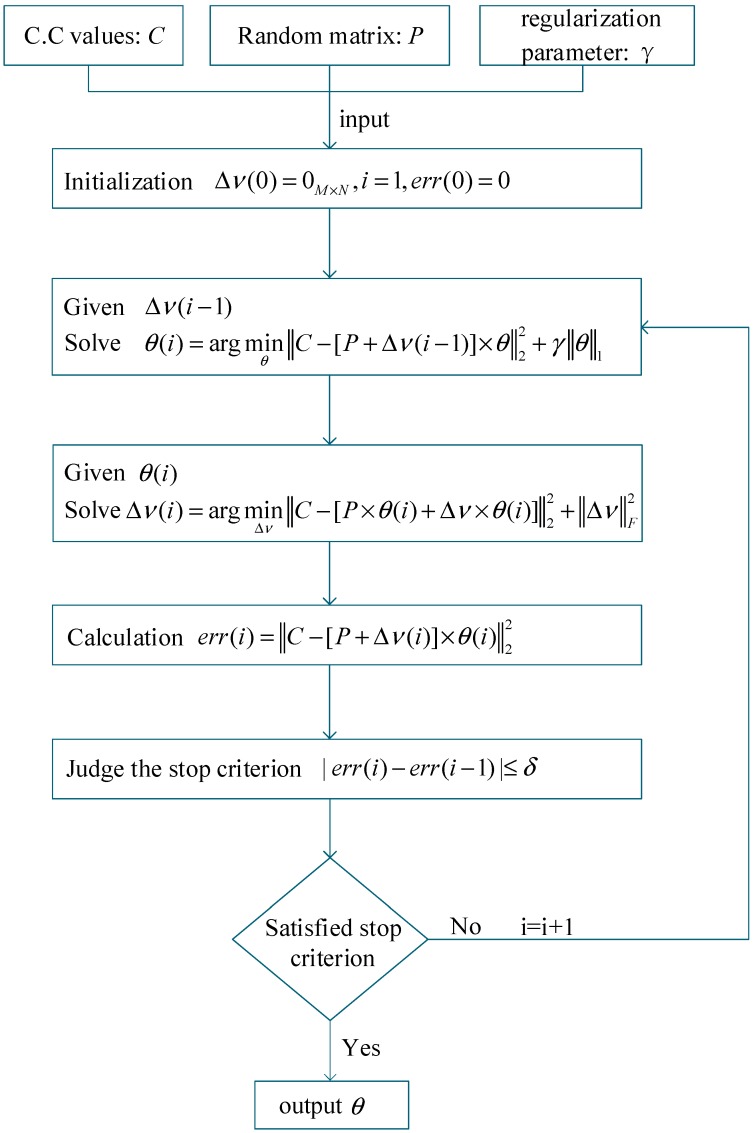
Coordinate descent method for solving STLS.

We use symlet wavelet in discrete wavelet transform (DWT), to transform the image into a sparse domain, then reconstruct the image with STLS algorithms in the sparse domain. We assume that the wavelet transform matrix is $W_{N\times N}$, then $W_{N\times N}\times T_{N\times 1}=\theta_{N\times 1}$, where $T_{N\times 1}$ is an unknown signal vector, and $\theta_{N\times 1}$ is the representation of $T_{N\times 1}$ in the sparse domain. Accordingly, Equation (12) can be rewritten as:

(13) $$\begin{array}{cc} C_{M\times 1} & =(A_{M\times N}+\Delta S)\times T_{N\times 1}+\Delta E\\ & =(A_{M\times N}+\Delta S)\times W_{N\times N}^{-1}\times \theta_{N\times 1}+\Delta E\\ & =(P_{M\times N}+\Delta \nu )\times \theta_{N\times 1}+\Delta E \end{array}$$

where $P_{M\times N}=A_{M\times N}\times W_{N\times N}^{-1}$ and $\Delta \nu =\Delta S\times W_{N\times N}^{-1}$. Then, we can obtain the flowchart of the STLS algorithm as shown in Figure 2, and the two steps are executed iteratively until an ideal solution is obtained.

Due to the errors existing in the model shown in Equation (8), the optimization problem can be formulated as:

(14) $$\begin{array}{l} \{\theta_{S-TLS},\Delta E_{S-TLS},\Delta \nu_{S-TLS}\}=\arg\underset{\Delta E,\theta ,\Delta \nu }{\min}\;{\left\|\left[\Delta E,\Delta \nu \right]\right\|}_{F}^{2}+\gamma {\left\|\theta \right\|}_{1}\\ s.t.\;C=(P+\Delta \nu )\theta +\Delta E \end{array}$$

the cost function includes two parts: the error term ${\left\|\left[\Delta E,\Delta \nu \right]\right\|}_{F}^{2}$ and the regularization term $\lambda {\left\|\theta_{1}\right\|}_{1}$ where γ is a parameter to control the sparsity of the solution. Clearly, when γ is equal to zero, the problem is reduced to the TLS. The optimization problem is non-convex. We set γ=300 and $\delta =1\times 10^{-10}$. We use a coordinate descent method to solve the problem. The method fixes a parameter between Δν and θ, while optimizing the other one, until a stop criterion is satisfied. The flowchart of the algorithm is illustrated in Figure 2. Once obtaining θ, we use $T_{N\times 1}=W_{N\times N}^{-1}\times \theta_{N\times 1}$ to transform θ to T, and then use *T* to obtain the reconstructed image.

## 3. Numerical Simulation Results

In order to examine the effectiveness of the proposed scheme with STLS, we compare the performance of the scheme with that of the scheme with OMP, GPSR, Method proposed in [19] and TLS algorithms. The method proposed in [19] is direct CS method. We use MSE and PSNR to evaluate the reconstruction quality. For an L×W image, the MSE and PSNR are defined as:

(15) $$MSE=\frac{\sum_{0\leq i<L}\sum_{0\leq j<W}{\left(C_{i,j}-C_{i,j}^{\prime }\right)}^{2}}{L\times W}$$

(16) $$PSNR=10\mathrm{lg}\frac{C_{max}^{2}}{MSE}$$

where $C_{i,j}$ represents the true image and $C_{i,j}^{\prime }$ denotes the reconstructed image, lg is the base-10 logarithm function, and $C_{max}$ is the maximum pixel value of the image. In our simulations, *L* = 64 and *W* = 64. Generally, the larger the PSNR, the better the quality of the reconstructed image. All the simulations are run using MATLAB 2014a in a computer with configuration: Intel(R) Core (TM) i7-7700 CPU 3.6 GHz and 16 GB memory.

**Figure 3:**
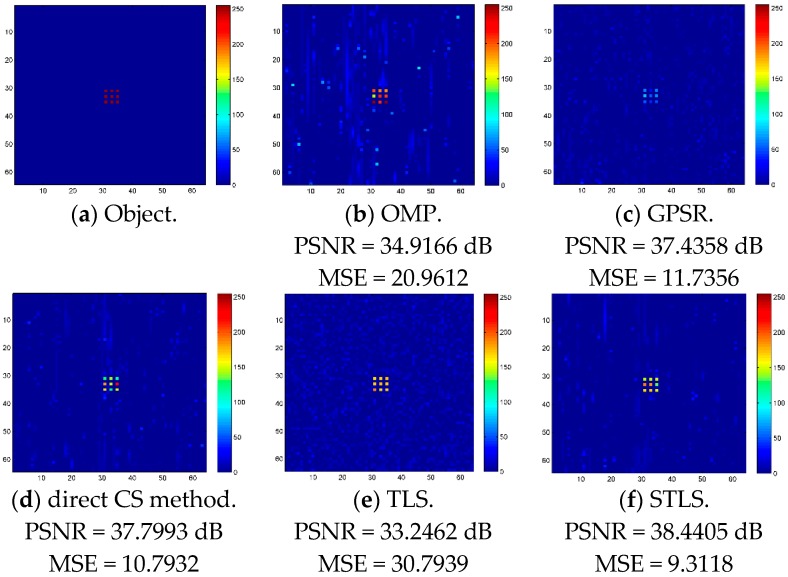
Reconstruction results of object with OMP, GPSR, Method proposed in [19], STLS (300 samples), and TLS (4500 samples). (**a**) Original object, (**b**) OMP result, (**c**) GPSR result, (**d**) Method proposed in [19], (**e**) TLS result and (**f**) STLS result.

In the simulations, we assume that the image scene includes 64 × 64 pixels, and there are nine objects in the scene as shown in Figure 3a, where each object is represented by a pixel and the interval between two adjacent objects is a pixel. This is used to evaluate the performance of the algorithm in the case of small objects. We added the disturbance ΔS and error ΔE into the image and assume that ΔS is Gaussian distributed with mean 0 and variance 0.1, and error ΔE in (12) is also Gaussian distributed with mean 0 and variance $0.5\times C_{max}$. The maximum intensity of the original image is 255.

**Figure 4:**
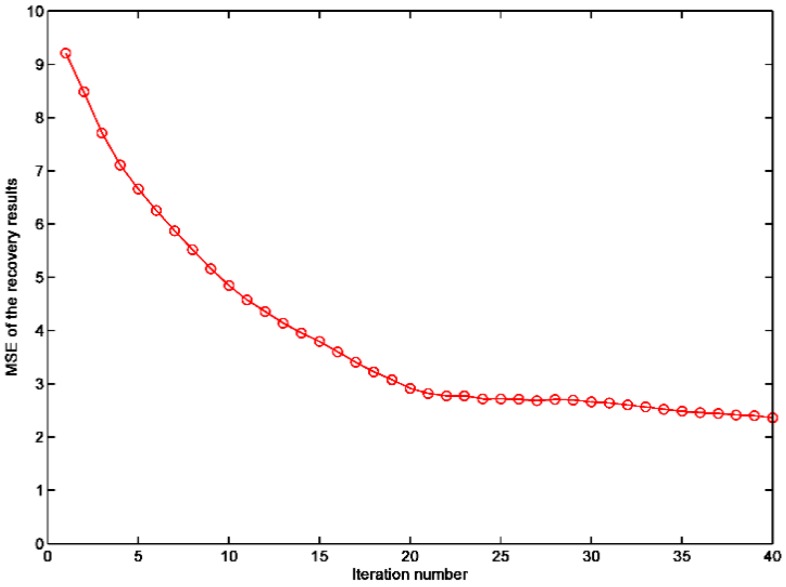
The MSE of the reconstruction image at different iterations when *λ* = 300, and the sampling number is 300.

The sampling number for OMP, GPSR, Method proposed in [19] and STLS is 300, and the sampling number for TLS is 4500 because TLS requires *M* > *N*. It can be seen in Figure 3 that OMP and TLS exhibit the worst performances; GPSR and Method proposed in [19] work slightly better. We can also see that STLS outperforms other algorithms significantly. Simulations show that the PSNRs of OMP, GPSR, Method proposed in [19], TLS and STLS are 34.9166 dB, 37.4358 dB, 37.7993 dB, 33.2462 dB and 38.4405 dB, respectively, and the MSEs of OMP, GPSR, Method proposed in [19], TLS and STLS are 20.9612, 11.7356, 10.7932, 30.7939 and 9.3118, respectively. STLS achieves much better PSNR and MSE than OMP, GPSR, Method proposed in [19] and TLS schemes. The convergence of the STLS algorithm and the typical execution times are shown in Figure 4 and Table 1, respectively. Figure 4 illustrates the MSE of the reconstruction image with different numbers of iterations. Normally, the algorithm converges within 20 iterations.

## 4. Experimental Results and Discussions

**Figure 5:**
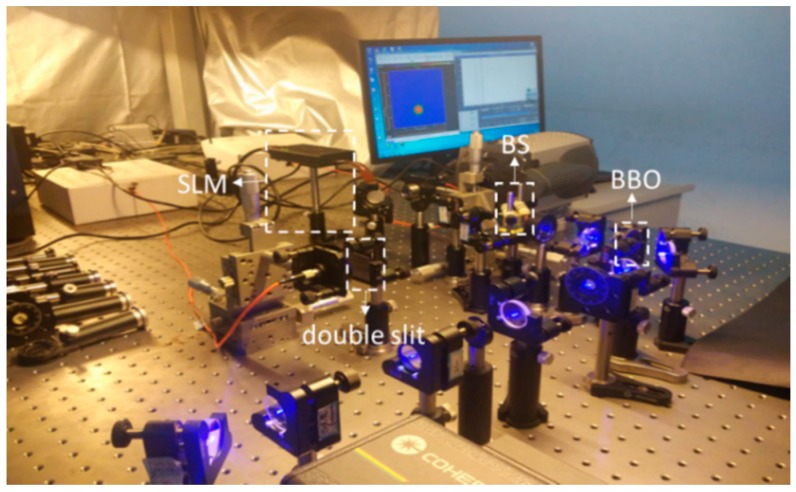
Experimental platform of our quantum GI, where random patterns are placed on the SLM, and the target is a double-slit.

Our quantum GI experimental system is shown in Figure 5. A continuous-wave laser with 460 nm wavelength was used to pump a BBO crystal, which was cut according to type-II collinear SPDC. The power of the pump laser is 300 mW, and the central wavelength and the bandwidth of the filter after BBO are 920 nm and 10 nm, respectively. The photon detection efficiency of single-photon detectors (SPCM-AQRH-FC, from Excelitas Technologies) at 920 nm is about 35%. In order to maximize the SPDC efficiency, we need to polarize the pump laser by a half-wave plate (HWP). Because only a small number of photons can be converted into entangled-photon pairs, we have to filter out the unconverted pump light by placing a filter behind BBO. A beam splitter (BS) was used to divide the entangled-photon pairs into the signal and reference arms. The entangled photons go through the object and are collected by the photon counting module (SPCM) in the object arm. The reference entangled photons are modulated by the SLM in the reference arm and are collected by the other SPCM. The SLM used in the experiment is HOLOEYE HES 6001-NIR with phase and amplitude type, and its resolution is 1920×1080 with pixel size 8×8 µm^2^.

**Figure 6:**
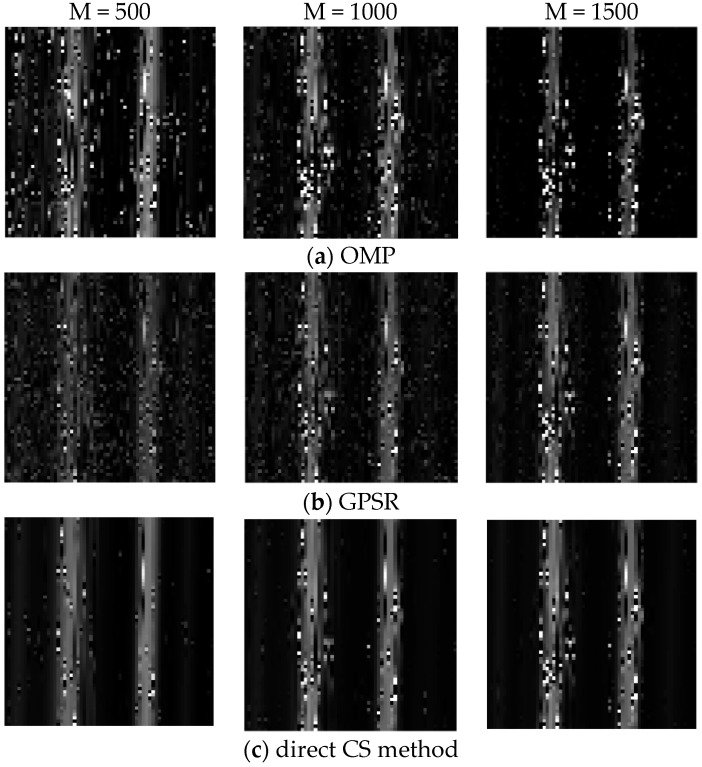

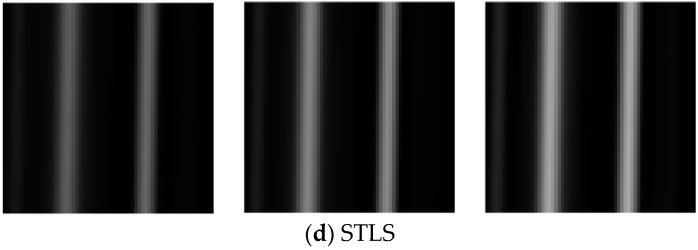
Experimental reconstructed quantum ghost images of the double-slit by (**a**) OMP, (**b**) GPSR, (**c**) Method proposed in [19] and (**d**) STLS.

To demonstrate the performance of STLS, we use double-slit as the object with 64×64 pixels shown in Figure 6. The singles counts we collected from SPCM in the object arm and reference arm are $5.05\times 10^{4}$ counts/s and $5.10\times 10^{4}$ counts/s. In the experiment, the center-to-center distance of the double-slit is 1200 µm (22 pixels) and the pixel pitch is 54 µm.

Figure 6 shows the reconstructed images by OMP, GPSR, Method proposed in [19] and STLS with sampling numbers of 500, 1000 and 1500, respectively. When the sampling number is 500, it is hard to identify the double-slit for OMP and GPSR. When the sampling number is 1000, we can see a blurry double slit in the images by OMP, GPSR and Method proposed in [19] but a clear one in the image by STLS, which demonstrates the advantage of the STLS scheme.

**Figure 7:**
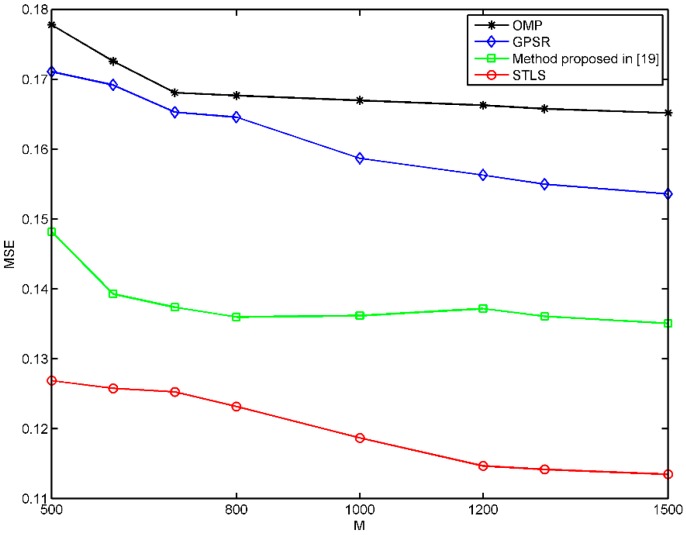
MSE of the reconstructed quantum ghost imaging with OMP, GPSR, Method proposed in [19] and STLS with different sampling numbers.

**Figure 8:**
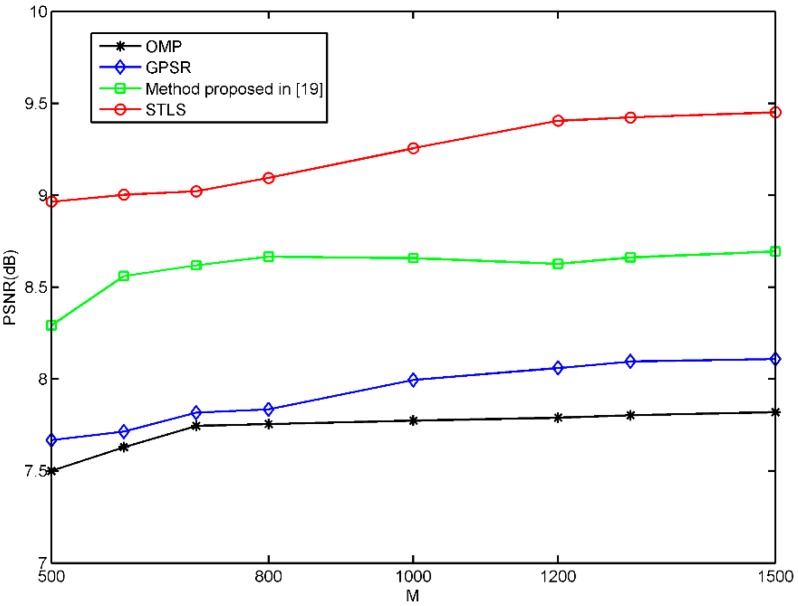
PSNR of the reconstructed quantum ghost imaging with OMP, GPSR, Method proposed in [19] and STLS with different sampling numbers.

PSNR and MSE were also used to evaluate the quality of the CS image reconstruction. Figure 7 and Figure 8 show the MSE and PSNR of three schemes, where we can see that the MSE of STLS is much smaller than the other two schemes, and the PSNR of STLS is much higher than the other two schemes. We can see that the STLS scheme achieves better reconstruction results than others with the same number of measurements. As the number of samples increases, better recovery results can be obtained. The performance of our quantum ghost imaging scheme can be optimized by improving the coincidence rate and the beam quality of the pump laser. In the experiment, the object has 64 × 64 = 4096 pixels. We can obtain a very high-quality image with 1500 measurements and a clear image with 1000 measurements.

## 5. Conclusions

In this work, we have investigated robust GI with a relatively small number of measurements to deal with the imperfection of optical devices, measurement error and noise in the optical path. The proposed method uses the pseudo-random matrix as the measurement matrix, and signals are transformed into a sparse domain by discrete wavelet transform. Then, the reconstruction is formulated as a sparse total least square problem which is solved iteratively. Both simulation and experimental results have been provided to show the superiority of the proposed STLS scheme, which can achieve significantly better PSNR and MSE than the system with other reconstruction algorithms such as TLS, OMP, GPSR and Method proposed in [19]. This work demonstrates the significant potential of handling the imperfection of optical devices in improving the quality of reconstruction.

## Figures and Tables

**Table 1:** The running time of each method.

Methods	OMP	GPSR	Method Proposed in [19]	STLS
Runtime	7.8582 s	29.5845 s	30.1480 s	102.5624 s
